# Nationwide study of emergency care quality for patients with substance use disorders and dual diagnoses across three distinct patient populations

**DOI:** 10.1186/s12888-025-06712-8

**Published:** 2025-03-31

**Authors:** Julie Mackenhauer, Mette Marie Berg, Søren Valgreen Knudsen, Erika F. Christensen, Jan Mainz, Søren Paaske Johnsen

**Affiliations:** 1 Department of Social Medicine, Aalborg University Hospital, Havrevangen 1, Aalborg, DK-9000 Denmark; 2 Danish Center for Health Services Research, Aalborg University, Selma Lagerløfs Vej 249, Gistrup, 9260 Denmark; 3 Department of Emergency Medicine, Regional Hospital Northern Denmark, Bispensgade 37, Hjørring, Denmark; 4 Centre for Prehospital and Emergency Research, Aalborg University Hospital, Søndre Skovvej 15, Aalborg, DK-9000 Denmark; 5 Psychiatry Northern Denmark Region, Mølleparkvej 10, Aalborg, DK-9000 Denmark

**Keywords:** Mental illness, Dual diagnosis, Substance use, Emergency care, Access to care, Prehospital care, Quality of care, Clinical registries

## Abstract

**Background:**

Substance use disorders and mental illness increase morbidity and mortality, particularly among patients with coexisting mental illness and substance use (dual diagnoses). This study evaluated the quality of prehospital care (Emergency Medical Services (EMS)) and emergency care for two time-dependent conditions among patients with mental illness, substance use disorders, and dual diagnoses.

**Methods:**

We analysed data from three nationwide Danish registries: 1) Danish Prehospital Registry (2016–2017), 2) Danish Stroke Registry (2010–2018), and 3) Danish Registry of Emergency Surgery (2008–2018), supplemented by national health and social registries. Quality of care was assessed using predefined metrics from the clinical registries. Exposure groups included patients with (a) mental illness, (b) substance use disorders, and (c) dual diagnoses, compared with a reference group without either diagnosis.

**Results:**

We identified 492,388 EMS calls, 89,148 admissions with ischemic stroke, and 3,223 emergency surgeries for perforated ulcers. Mental illness, substance use disorders, and dual diagnoses were most prevalent in the EMS cohort (10%, 9%, and 8%, respectively).

Compared with reference patients, EMS patients with mental illness, substance use, or dual diagnoses were more likely to make repeat EMS calls within 24 h (RR 1.60 [1.39–1.83], 2.32 [2.02–2.66], and 3.24 [2.89–3.53]) and have unplanned hospital visits within seven days after EMS-release at scene, i.e. patient weas not transported to the hospital (scene release) (RR 1.50 [1.39–1.62], 1.58 [1.45–1.73], and 2.50 [2.31–2.71]).

Stroke patients with mental illness, substance use, or dual diagnoses were less likely to receive reperfusion therapy for ischemic stroke (RR 0.80 [0.74–0.86], 0.60 [0.54–0.66], and 0.69 [0.60–0.80]) but had rates of other guideline-based stroke care like the reference group without mental illness or substance abuse.

Compared with the reference population, patients with perforated ulcers and mental illness experienced a longer time to surgery, with a delay of 82 min (95% CI: 37–128). Within the first 90 days after surgery, patients were 69 days (IQR [0;83]) alive-and-out-of-hospital; however, patients with mental illness, substance use and dual diagnoses lost a median of 4 days (IQR [-1;9]), 6 days [1;10], and 7 days [0;13], respectively, due to early mortality compared with the reference.

**Conclusions:**

Disparities in prehospital and emergency care were identified across three distinct cohorts: a broad EMS population and two time-critical conditions. Patients with mental illness, substance use disorders, and dual diagnoses faced inequities in EMS response, reperfusion therapy, surgical timeliness contributing to poorer short-term outcomes. However, areas of consistent care quality were observed, particularly in guideline-based stroke care.

**Supplementary Information:**

The online version contains supplementary material available at 10.1186/s12888-025-06712-8.

## Background

People with substance use disorders, particularly those who also have mental health conditions (dual diagnosis), face substantial barriers when seeking care [1, 2]. Studies have shown that healthcare systems often lack integration between mental health and substance use treatments, creating care gaps and leaving needs unmet [3, 4]. This lack of coordination can drive individuals to rely more on emergency services for immediate support rather than seeking care through primary or community healthcare systems [4–6]. Barriers such as limited access to primary care, poor continuity of care, and challenges in navigating healthcare pathways exacerbate this reliance, particularly for patients with mental illness, substance use disorders, or dual diagnoses [4, 5].

For those living with dual-diagnosis challenges, the burden of disease is high, including more years spent with disability and reduced life expectancy [7–9]. Untreated mental health conditions add significant costs to the healthcare system, and research shows that these patients tend to require more frequent healthcare services, especially when ongoing care options are limited [8, 10].

Integrated care and tailored interventions addressing both mental health and substance use are increasingly recognized as essential for improved outcomes. Bahji (2024) highlights collaborative care models that integrate primary care, mental health, and addiction services to improve treatment adherence and access for dual-diagnosis patients [11]. Sweileh (2024) described evidence-based approaches such as “Integrated Dual Diagnosis Treatment”, which treats mental health and substance use disorders simultaneously, and “Assertive Community Treatment (ACT)”, a community-based model that reduces hospitalizations and enhances rehabilitation [12]. While these models address care fragmentation and benefit high-need populations, further research is needed to assess their impact on the reliance of emergency care.

Tailored primary care interventions show promise in reducing emergency care dependency: Tranberg & Colnadar (2024) highlight multidisciplinary teams with mental health and addiction expertise [13], whereas Sweileh (2024) describes the Patient-Centred Medical Home (PCMH) model, which integrates these services to improve accessibility and continuity of care [12]. These approaches, along with efforts such as targeted provider training to address stigma, [10] underscore the need for scalable, evidence-based strategies to better meet the needs of patients with cooccurring mental health and substance use challenges.

Taken together, these findings highlight the need for care models that genuinely address the unique needs of those with mental health and substance use challenges. Improved coordination of services may help support well-being and create a more responsive, efficient healthcare system [1, 4, 9].

Previous research highlights considerable disparities in care for individuals with mental health and substance use challenges, particularly in long-term care settings [4, 12]. While the effects of fragmented care are well documented—including delayed treatment and poorer health outcomes—their specific challenges in emergency settings remain less understood. Addressing these gaps is essential for developing focused interventions that provide more effective acute care for this population.

### Objective

This study aims to evaluate the quality of prehospital and emergency care across three distinct populations: a broad cohort of patients using the prehospital/emergency medical services (EMS) and two condition-specific cohorts requiring time-dependent emergency care (ischaemic stroke and perforated ulcers). Disparities in process measures and clinical outcomes among patients with substance use disorders, psychiatric conditions, and dual diagnoses were compared to those of a reference population without these conditions.

## Methods

### Study design

This study includes data from three nationwide Danish clinical registries: 1) Danish Prehospital Registry (2016–2017), 2) Danish Stroke Registry (2010–2018), and 3) Danish Registry of Emergency Surgery (2008–2018). It utilizes comprehensive healthcare data to investigate disparities in care and outcomes among individuals with mental illness, substance use disorders, and dual diagnoses. The study covers a 3–11-year period, depending on the availability of the selected variables in the clinical registries (Table 1). The study was reported according to the STROBE guidelines [14].

**Table 1:** Selected outcome measures from three Danish clinical registries nationwide

Outcome Measure	Definition	Data Source	Years
Repeated EMS contact	Repeated EMS call within 24 h following initial telephone advice	Danish Quality Registry for Prehospital EMS	2016–2017
Unplanned hospital visits^a^	Unplanned hospital contacts within 7 days after scene release *(i.e. EMS patient was not transported to the hospital* = *scene release)* ^a^	Danish Quality Registry for Prehospital EMS^a^	2016–2017
Reperfusion therapy	Receiving thrombolysis or thrombectomy for ischemic stroke	Danish Stroke Registry	2010–2018
CT/MR imaging	Patient is examined with a CT/MRI scan on the day of admission	Danish Stroke Registry	2010–2018
Early mobilization	Patient is mobilized on the day of admission	Danish Stroke Registry	2010–2018
Indirect swallowing test	Patient receives an indirect swallowing test on the day of admission to assess swallowing function and risk of aspiration before receiving food or fluids	Danish Stroke Registry	2011–2018
Direct swallowing test	Patient receives a direct swallowing test on the day of admission to assess swallowing function and risk of aspiration before receiving food or fluids	Danish Stroke Registry	2011–2018
Physiotherapy	Patient is assessed by a physiotherapist within the second day of admission to determine rehabilitation needs (type and extent)	Danish Stroke Registry	2010–2018
Occupational therapy	Patient is assessed by an occupational therapist within the second day of admission to determine rehabilitation needs (type and extent)	Danish Stroke Registry	2010–2018
Nutrition screening	Patient receives a nutritional risk assessment within the second day of admission	Danish Stroke Registry	2010–2018
Stroke unit admission	Patient is admitted to a stroke unit within the second day of admission	Danish Stroke Registry	2010–2018
Platelet inhibitor therapy	Patient with ischemic stroke without atrial fibrillation receives platelet inhibitor therapy within the second day of admission	Danish Stroke Registry	2010–2018
Oral anticoagulation therapy	Patient with ischemic stroke and atrial fibrillation receives oral anticoagulation therapy within 14 days of admission	Danish Stroke Registry	2010–2018
Recurrent stroke	Incidence of a new stroke event within one year after initial stroke admission	Danish Stroke Registry	2010–2018
30-day stroke mortality	Death within 30 days after hospital admission for ischemic stroke	Danish Stroke Registry	2010–2018
Time-to-surgery	Time in hours from hospital arrival to surgery for patients with perforated peptic ulcers	Danish Registry of Emergency Surgery	2008–2018
Days-alive-and-out-of-hospital	Total number of days the patient is alive and not hospitalized within 90 days postsurgery	Danish Registry of Emergency Surgery	2008–2018
30-day surgical mortality	Death within 30 days of the emergency surgical procedure	Danish Registry of Emergency Surgery	2008–2018

^a^ Measure was defined by the authors of this and previous papers, rather than the clinical registry [17]

### Setting

This study was conducted in Denmark, which has a tax-funded healthcare system providing universal access to healthcare services. The system is organized into five regions that oversee hospitals, psychiatric care, general practitioners (GPs), and emergency medical services (EMSs). In a Danish setting, the EMS is defined by prehospital care provided by ambulance services and emergency call centres. This concerns all medical 112-calls in a Danish setting, Funding is provided primarily through national taxes, with the regions responsible for administering hospital services and the municipalities handling social care and rehabilitation, including community-based addiction treatment. Danish citizens have a unique Civil Personal Register (CPR) number, allowing the linkage of health data across registries and supporting the integration of services.

### Participants

Patients were identified based on their inclusion in one of the three clinical registries (Table 1) and categorized into four groups based on exposure. Mental illness was defined broadly but includes heterogeneous conditions with varying severity. To assess whether these differences influence our findings, we conducted supplementary subgroup analyses distinguishing major mental illness (e.g., schizophrenia, bipolar disorder) from moderate mental illness (e.g., depression, anxiety disorders). Similarly, we examined whether outcomes differed between patients with alcohol vs. drug-related substance use disorders (see Supplementary Material).

1. Current or recent substance use: Registered in the National Registry of Alcohol Treatment [15] or the Register of Substance Abusers in Treatment [16] *or* with an ICD-10 diagnosis of mental and behavioural disorders due to psychoactive substance use (DF10-F19) *within 5 years.*
2. Current, recent or history of mental illness: While the authors of this paper previously described the quality of emergency care for patients with a history of “major”, “moderate” or “minor” mental illness, [17] we defined mental illness as
  - *Within 10 years:* A hospital-based diagnosis of schizophrenia spectrum disorders (DF20-22), bipolar disorder (DF30-31), *or* admission (> 2 days) with depression (DF32-34) or emotionally unstable personality disorder (DF60.3) *or*
  - *Within 5 years*: any other psychiatric diagnoses (DF23-99) with or without admission *or* with any private psychiatrist consultation*.*
3. Dual diagnosis: Individuals who meet the criteria for both substance use and mental health conditions.

Reference: Individuals without registrations related to substance use or mental health conditions.

### Outcomes (for overview – see Table 1):

- Prehospital Care: Repeated EMS contact within 24 h, and unplanned hospital visits within 7 days of scenerelease (i.e. EMS patient was not transported to the hospital)
- Stroke Care: Proportion of patients receiving reperfusion therapy, provision of guideline-based acute stroke care (ten measures), recurrent stroke within one year, and 30-day mortality.
- Emergency surgery: Time-to-surgery, days alive and out of the hospital within 90 days postsurgery.

### Other variables

Covariates available for all patients included age, sex, comorbidities (Charlson Comorbidity Index 0, 1–2, 3–4, or 5 +), smoking status (never smoker, previous smoker, or current smoker), socioeconomic position (personal income (% living in relative poverty according to the national consensus definition), level of education (high/medium/low), adherence to the workforce (% receiving public benefits), and cohabitation status (% living alone). For patients with ischaemic stroke, stroke severity (Scandinavian stroke scale), time of hospital arrival (regardless of arrival time at the stroke unit), and onset of stroke symptoms (known/unknown/last known well) were available. For EMS patients, symptoms (chief complaint according to the “Danish Index”) were available. The American Society of Anaesthesiologists (ASA) classification score is available for emergency surgical patients. Please see Supplement material Table 1 for data sources and definition of co-variates.

### Potential confounders

Please see the Supplementary Material for the Directed Acyclic Graph (DAG) illustrating the classification of covariates as confounders, mediators, or unmeasured variables. We adjusted for key confounders, including age, sex, and comorbidities, via the Charlson Comorbidity Index. For ischemic stroke, we additionally adjusted for stroke severity using the Scandinavian Stroke Scale, as it influences clinical outcomes and emergency care quality. However, we acknowledge that stroke severity could also act as a mediator if mental illness or substance use indirectly affects severity at admission (e.g., through delayed care-seeking). We chose this approach to ensure consistency with existing literature and comparability across studies. Socioeconomic factors, such as income and education, were treated as mediators rather than confounders. While socioeconomic factors may shape mental health, substance use status and health outcomes, they may even more likely act as mediators between exposure and health outcomes and was treated accordingly. For transparency, we presented unadjusted estimates, Model 1 (adjusted for age and sex), and Model 2 (adjusted for age, sex, comorbidities, and stroke severity for stroke analyses). This strategy allows readers to assess the impact of different adjustments on our findings.

### Data sources

Data were extracted from the Danish Quality Registry for Prehospital Emergency Medical Services [18] (2016–2017), the Danish Stroke Registry [19] (2010–2018), and the Danish Registry of Emergency Surgery (2008–2018) (Table 1). While data from the Stroke Registry and Register for Emergency Surgery were available before 2010, data on substance use treatment were only available from 2008 onwards; however, most patients with substance use were identified through ICD-10 codes rather than municipality-based treatment. With the selected exposure period of two years prior to admission, we restricted the outcome variables to be from 2010 onwards.

The Danish Stroke Registry is a validated nationwide clinical quality database with an estimated sensitivity of 97% [20]. Only patients registered with an acute ischemic stroke (International Classification of Diseases, Tenth Revision DI63.x-64.x) were included. Although DI64 is defined as “Stroke, not specified as haemorrhage or infarction,” all patients included in the Danish Stroke Registry had relevant imaging (computed tomography/magnetic resonance) performed to exclude haemorrhage.

### Study size

The study size was determined by the number of eligible patients recorded in the registries during the study periods (2016–2017, 2010–2018 and 2008–2018). Given the use of nationwide registry data, the sample size reflects the entire population meeting the study criteria within Denmark during the specified time frame.

### Statistical methods

We analysed data from three distinct populations (EMS, ischemic stroke, and emergency surgery for perforated ulcers) via robust statistical methods tailored to each outcome. For binary outcomes, such as reperfusion therapy or repeat EMS contact, we calculated risk ratios (RRs) via modified Poisson regression. Continuous outcomes, such as time-to-surgery and days alive-and-out-of-hospital, were analysed via robust linear regression based on Huber and bi-weight iterations to limit the influence of outliers. Missing values for the time of surgery were imputed by implementing multiple imputation chained equations via 20 imputation sets. For all analyses, unadjusted and adjusted estimates were calculated. Two models were adjusted: Model 1 was adjusted for age and sex, Model 2 also included comorbidities. For stroke patients Model 2 further included stroke severity score (Scandinavian Stroke Severity Scale). Age was modelled as restricted cubic splines with three knots at quantiles of 0.1, 0.5, and 0.9 to capture nonlinear effects.

Sensitivity analyses were conducted to test key outcomes, specifically, whether both “major” and “moderate” mental illness should be included in the definition of “mental illness”, as well as differentiating substance use based on an ICD-10 diagnose, alcohol treatment and opioid-replacement therapy, respectively. Furthermore, analyses for reperfusion therapy were restricted to patients arriving within 4 h from the onset of symptoms.

All the statistical analyses were performed via Stata (version 16; StataCorp, College Station, TX), ensuring consistency and rigor across the three cohorts.

## Results

In three distinct populations, we identified 492,388 EMS calls (2016–2017), 89,148 admissions with ischemic stroke (2010–2018), and 3,223 emergency surgeries for perforated ulcers (2008–2018). The proportions of patients with a history of mental illness, substance use disorders, and dual diagnoses differed across these populations: 10%, 9%, and 8% of EMS encounters, respectively, compared to 5%, 4%, and 2% of ischemic stroke admissions, and 5%, 9%, and 3% of perforated ulcer cases (Table 2).

**Table 2:** Baseline characteristics of the three populations

	**Prehospital/EMS** **2016–2018** **(n = 492,388)**				**Ischemic stroke** **2010–2018** **(n = 89,148)**				**Emergency surgery – perforated ulcer** **2008–2018** **(n = 3,223)**			
	**None**	**Mental** **illness**	**Substance** **use**	**Dual**	**None**	**Mental** **illness**	**Substance** **use**	**Dual**	**None**	**Mental** **illness**	**Substance** **use**	**Dual**
**Number and proportion (%)**	358,206 (73%)	49,427 (10%)	43,379 (9%)	41,376 (8%)	79,689 (89%)	4,726 (5%)	3,410 (4%)	1,323 (2%)	2,628 (83%)	176 (5%)	273 (9%)	92 (3%)
**Sex (Male %)**	183,101 (51%)	19,618 (40%)	30,251 (70%)	24,800 (60%)	43,373 (55%)	2,013 (43%)	2,298 (70%)	839 (63%)	1,216 (45%)	72 (41%)	163 (60%)	54 (59%)
**Age, mean IQR**	68 y [43;80]	51 y [29;70]	57 y [45;68]	49 y [35;59]	75 y [65;83]	70 y [59;81]	67 y [58;74]	61 y [53;69]	73 y [62;82]	66 y [54;77]	60 y [50;70]	61 y [49;68]
**Comorbidity index (CCI)** **0**	169,571 (47%)	26,196 (53%)	16,760 (39%	19,058 (46%)	2,435 (3%)	167 (4%)	94 (3%)	35 (3%)	116 (4%)	12 (7%)	9 (3%)	2 (2%)
**1–2**	111,744 (31%)	14,508 (19%)	14,800 (34%)	14,034 (35%)	53,054 (67%)	2,977 (63%)	1,921 (56%)	800 (60%)	1,713 (64%)	115 (65%)	139 (51%)	51 (55%)
**3–4**	46,146 (13%)	5,452 (11%)	6,185 (14%)	4,666 (11%)	16,511 (21%)	1,052 (22%)	812 (24%)	305 (23%)	546 (20%)	36 (20%)	67 (25%)	20 (22%)
**5 + **	30,745 (9%)	3,271 (7%)	5,633 (13%)	3,618 (9%)	7,689 (10%)	530 (11%)	583 (17%)	182 (14%)	307 (111%)	13 (7%)	58 (21%)	19 (21%)
**Smoking** **Never**	N/A				26,381 (38%)	1,351 (34%)	298 (10%)	110 (9%)	847 (33%)	49 (30%)	17 (7%)	9 (10%)
**Previous**					22,410 (32%)	1,065 (27%)	604 (20%)	195 (16%)	456 (18%)	25 (10%)	27 (10%)	7 (8%)
**Current**					20,775 (30%)	1,601 (40%)	2,174 (71%)	901 (75%)	1,228 (49%)	90 (55%)	217 (83%)	74 (82%)
**Living alone, proportion (%)**	164,609 (47%)	31,841 (65%)	31,317 (73%)	34,389 (83%)	36,747 (46%)	3,001 (64%)	2,189 (64%)	964 (73%)	1,457 (55%)	115 (65%)	178 (66%)	72 (78%)
**Level of education High**	60,624 (18%)	7,042 (15%)	4,504 (11%)	4,263 (11%)	12,921 (17%)	791 (18%)	391 (12%)	200 (16%)	322 (13%)	25 (15%)	25 (10%)	17 (19%)
**Medium**	126,506 (38%)	14,881 (31%)	15,313 (37%)	12,166 (31%)	29,793 (39%)	1,588 (36%)	1,420 (43%)	520 (40%)	899 (36%)	59 (35%)	101 (39%)	40 (44%)
**Low**	143,897 (44%)	25,435 (54%)	21,588 (52%)	23,127 (60%)	32,729 (43%)	2,077 (47%)	1,466 (45%)	566 (44%)	1,252 (51%)	84 (50%)	133 (51%)	33 (37%)
**Personal income, relative** **Poverty (%)**	20,876 (6%)	6,490 (14%)	6,159 (14%)	8,746 (21%)	1,441 (2%)	135 (3%)	160 (5%)	72 (5%)	87 (3%)	8 (5%)	17 (6%)	10 (11%)
**No attachment to workforce, Public benefits (%)**	44,073 (13%)	20,368 (44%)	20,793 (49%)	29,808 (73%)	7,116 (9%)	1,276 (27%)	1,111 (33%)	708 (54%)	356 (14%)	62 (36%)	121 (45%)	56 (61%)
**ASA score** **1**	Not relevant				Not relevant				438 (16%)	33 (19%)	23 (8%)	5 (5%)
**2**									954 (36%)	60 (35%)	83 (31%)	30 (33%)
**3**									950 (36%)	60 (35%)	124 (46%)	45 (50%)
**4–5**									313 (12%)	21 (11%)	40 (25%)	11 (12%)
**Stroke severity,** Scandinavian Stroke Scale (SSS) **Mild** **Moderate** **Severe** **Very Severe**	Not relevant				51,092 (66%)	1,802 (62%)	2,024 (62%)	822 (65%)	Not relevant			
					14,045 (18%)	952 (21%)	721 (22%)	274 (22%)				
					6,621 (9%)	444 (10%)	330 (10%)	106 (8%)				
					5,294 (7%)	325 (7%)	205 (6%)	59 (5%)				
**Time onset of symptoms to hospital arrival, minutes [IQR]**	N/A				315 min [110; 1120]	330 min [115; 1135]	378 min [120; 1422]	347 min [111; 1246]	N/A			
**Time of symptom onset known,** Yes (%)	N/A				65,981 (83%)	2,767 (80%)	2,695 (79%)	1,016 (77%)	N/A			
**Presenting symptom,** **4 most common, proportion (%)** *(“Danish Index”)*	Unclear problem (15%) Chest pain (15%) Impaired consciousness (12%) Other accident (not traffic) 11%)	Unclear problem (15%) Chest pain (14%) Other accident (not traffic) (9%) Impaired consciousness (9%)	Unclear problem (18%) Alcohol, intoxication, overdose (11%) Other accident (not traffic) 11%) Chest pain (10%)	Unclear problem (18%) Alcohol, intoxication, overdose (15%) Psychiatry or suicide (10%) Chest pain (9%)	N/A				N/A			

Across all populations, exposed groups differed significantly from the reference population. Patients with mental illness, substance use disorders, or dual diagnoses were generally younger, had lower income levels, were more likely to live alone, and were more frequently reliant on public benefits. Additionally, males were more common among those with substance use disorders and dual diagnoses than in the reference groups (Table 2).

### EMS Population

**Fig. 1:**
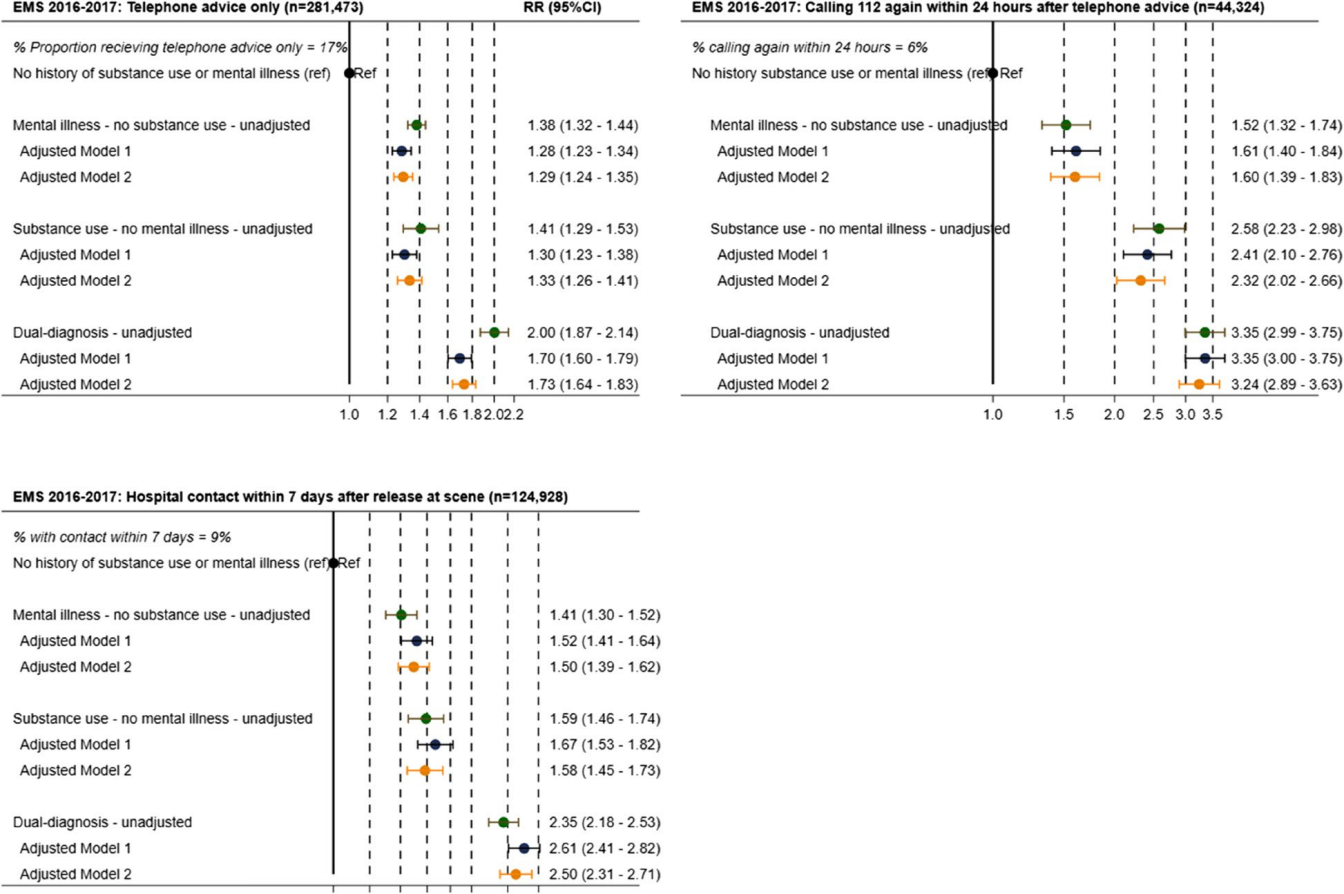
Forest plots of EMS outcomes by exposure group: risk ratios (RR) in the EMS population (2016–2017): (1) receiving telephone advice only, (2) making a repeat EMS call within 24 h following telephone advice, and (3) having unplanned hospital contact within 7 days after release at the scene. The results are shown for four exposure groups: no history of mental illness or substance use disorders (reference group), (a) mental illness, (b) substance use disorder, and (c) dual diagnoses (cooccurring mental illness and substance use disorders). Each plot includes unadjusted RRs, as well as RRs adjusted for age and sex (adjusted model 1), and further adjusted for comorbidity (adjusted model 2)

In prehospital care, 17% of all EMS calls resulted **in** telephone advice rather than ambulance dispatch. Patients with mental illness, substance use disorders, or dual diagnoses were more likely to receive telephone advice, with adjusted risk ratios of 1.29 (95% CI: 1.24–1.36), 1.33 (1.26–1.41), and 1.73 (1.64–1.83), respectively, compared to the reference population (Fig. 1). The likelihood of repeat EMS calls within 24 h was higher in the exposed groups, at 6% for patients with mental illness, 12% for those with substance use disorders, and 15% for dual-diagnosis patients, compared to 4% in the reference group. Adjusted risk ratios for repeat EMS calls were 1.60 (1.39–1.83), 2.32 (2.02–2.66), and 3.24 (2.89–3.53), respectively (Fig. 1). Among those released at the scene, unplanned hospital contacts within seven days occurred in 10%, 12%, and 19% of patients with mental illness, substance use disorders, and dual diagnoses, respectively, compared to 7% in the reference group, corresponding to adjusted risk ratios of 1.50 (1.39–1.62), 1.58 (1.45–1.73), and 2.50 (2.31–2.71) (Fig. 1).

### Ischemic stroke population

**Fig. 2:**
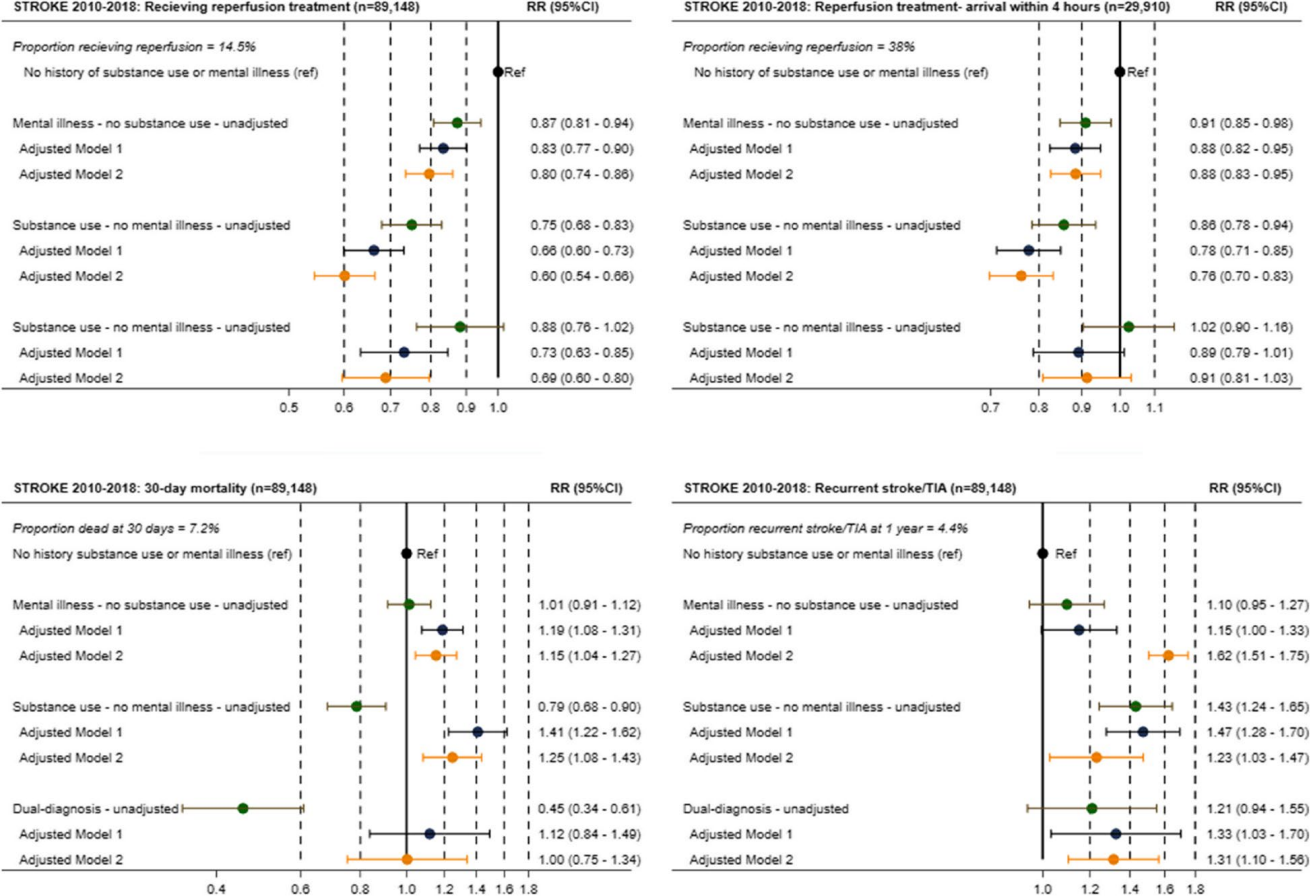
Forest plots of stroke outcomes by exposure group. Risk ratios (RRs) for key outcomes in the stroke population (2010–2018): (1) receiving reperfusion therapy, (2) receiving reperfusion therapy restricted to patients arriving within 4 h of symptom onset, (3) 30-day mortality, and (4) recurrent stroke within 1 year. The results are shown for four exposure groups: no history of mental illness or substance use disorders (reference group), (a) mental illness, (b) substance use disorder, and (c) dual diagnoses (cooccurring mental illness and substance use disorders). Each plot includes unadjusted RRs, as well as RRs adjusted for age and sex (Model 1), and further adjusted for the Charlson comorbidity index and stroke severity (Model 2)

**Fig. 3:**
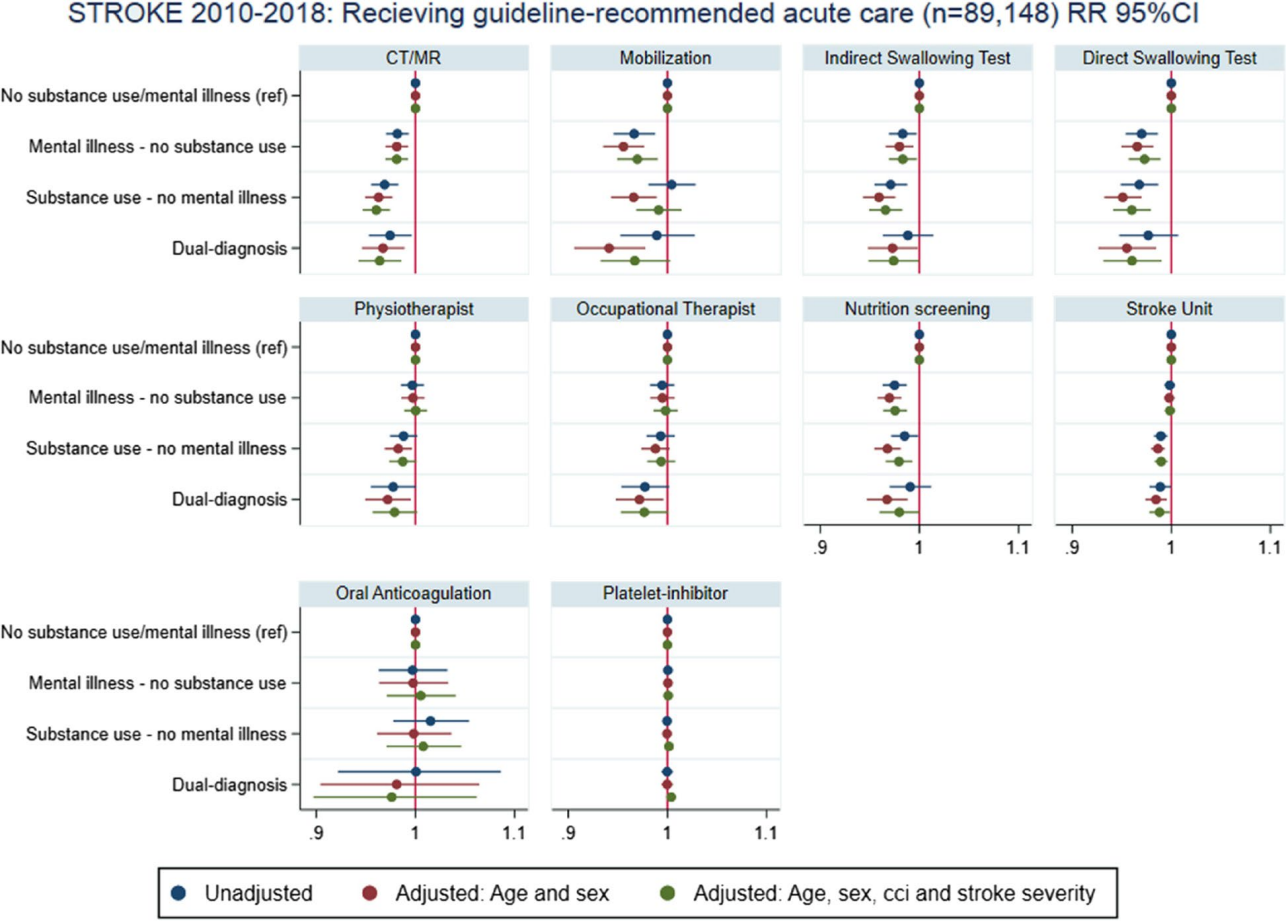
Forest plots of guideline-based stroke care by exposure group: (RRs) for process measures in the stroke population (2010–2018) including (1) CT/MRI imaging on the day of admission, (2) mobilization on the day of admission, (3) indirect swallowing test on the day of admission, (4) direct swallowing test on the day of admission, (5) physiotherapy assessment within the second day of admission, (6) occupational therapy assessment within the second day of admission, (7) nutrition screening within the second day of admission, (8) admission to a stroke unit within the second day of admission, (9) treatment with platelet inhibitors for patients without atrial fibrillation within the second day of admission, and (10) oral anticoagulation therapy for patients with atrial fibrillation within 14 days of admission. The results are shown for four exposure groups: no history of mental illness or substance use disorders (reference group), (a) mental illness, (b) substance use disorder, and (c) dual diagnoses (cooccurring mental illness and substance use disorders). Each plot includes unadjusted RRs, as well as RRs adjusted for age and sex (Model 1), and further adjusted for the Charlson comorbidity index and stroke severity (Model 2)

Among the 89,148 ischemic stroke admissions, 14.5% of patients received reperfusion therapy. Patients with mental illness, substance use disorders, or dual diagnoses were less likely to receive reperfusion therapy, with rates of 14%, 12%, and 14%, respectively, compared to 16% in the reference group. Adjusted risk ratios were 0.80 (0.74–0.86), 0.60 (0.54–0.66), and 0.69 (0.60–0.80), respectively (Fig. 2). When restricted to patients arriving within four hours of symptom onset, these differences remained significant (Fig. 2). Other guideline-based stroke care measures, including CT/MRI imaging, stroke unit admission, and supportive therapies such as physiotherapy, occupational therapy, and nutritional screening, were provided at similar rates across groups, with minor statistical differences deemed clinically negligible (Fig. 3).

The overall 30-day poststroke mortality rate was 7%, with higher adjusted mortality observed in patients with mental illness (RR 1.15 [1.04–1.27]) and substance use disorders (RR 1.25 [1.08–1.43]), but no significant difference for dual diagnoses (RR 1.00 [0.75–1.34]) (Fig. 2). Recurrent stroke within one year was more common in patients with mental illness (RR 1.62 [1.51–1.75]), substance use disorders (RR 1.23 [1.03–1.47]), and dual diagnoses (RR 1.31 [1.10–1.56]) when adjusted for age, sex, comorbidity, and stroke severity (Fig. 2).

### Emergency surgery population

**Fig. 4:**
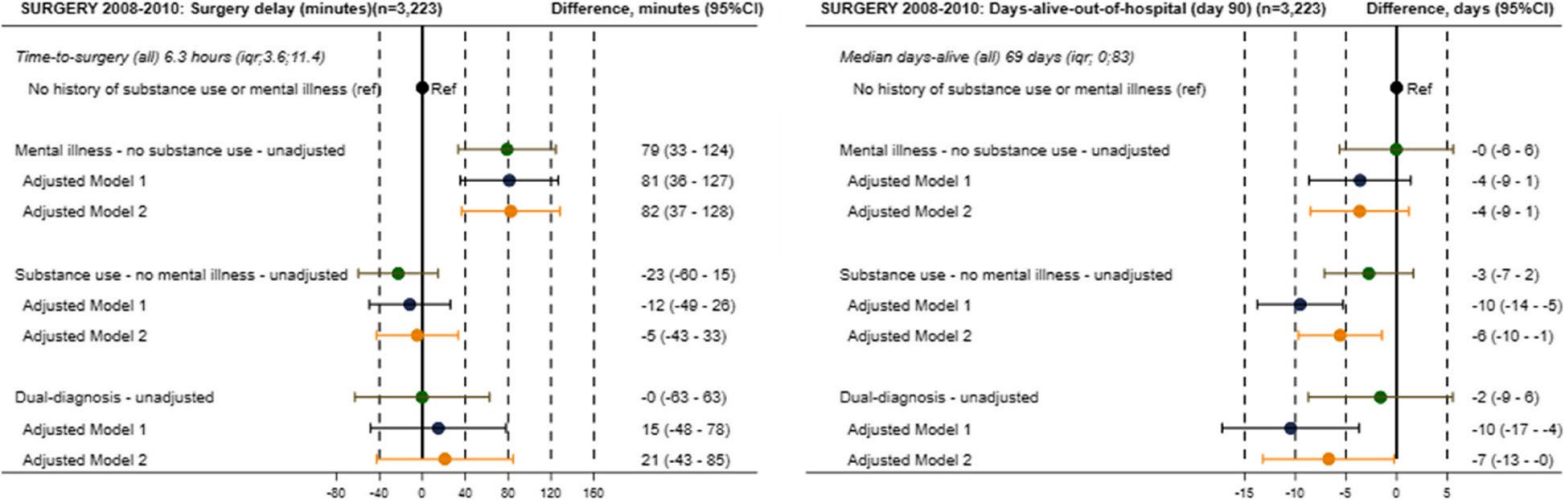
Forest plots of surgical outcomes by exposure group. This figure presents differences in key outcome measures for the surgical population (2008–2018). 1) Time-to-surgery, defined as the time in hours from hospital arrival to surgery for patients with perforated peptic ulcers, and 2) Days alive and out of hospital, calculated as the total number of days patients are alive and not hospitalized within 90 days postsurgery. The results are shown for four exposure groups: no history of mental illness or substance use disorders (reference group), (a) mental illness, (b) substance use disorder, and (c) dual diagnoses (cooccurring mental illness and substance use disorders). Adjusted differences are presented for both age- and sex-adjusted models (Model 1), and the models are further adjusted for comorbidities (Model 2)

The median time-to-surgery for all patients with perforated ulcers was 6.3 h. Patients with mental illness experienced delays, with a median time-to-surgery of 7.5 h compared to 6.1 h in the reference group, reflecting an adjusted delay of 82 min (95% CI: 37–128) (Fig. 4). Patients with substance use disorders or dual diagnoses did not experience significant surgical delays. Within the first 90 days post-surgery, patients with mental illness, substance use disorders, and dual diagnoses had median days alive and out of the hospital of 71, 61, and 63 days, respectively, compared to 70 days in the reference group. Adjusted differences showed a median loss of 4 days (IQR [−1;9]) for patients with mental illness, 6 days (IQR [1;10]) for those with substance use disorders, and 7 days (IQR [0;13]) for those with dual diagnoses, reflecting early mortality and prolonged hospital stays (Fig. 4).

### Sensitivity analyses

When restricting “mental illness” to major mental illness (e.g., schizophrenia, bipolar disorder), effect sizes for mental illness increased, particularly in the EMS population, while differences for substance use disorders and dual diagnoses diminished (supplementary Fig. 2). Findings for ischemic stroke and surgery remained largely unchanged (not presented). These results indicate that moderate mental illness contributes to poorer outcomes and support its inclusion in the dual-diagnosis definition. Previous investigations by the authors have detailed the contributions of different severity levels of mental illness to emergency care disparities [17–19, 21–23]. When analyzing the three subgroups defining 'substance use' (ICD-10 diagnosis, alcohol treatment, and opioid replacement therapy), all demonstrated a higher risk of poor i.e. prehospital outcomes (Supplementary Fig. 3). While the substance use group is heterogeneous, the consistently elevated risk across subgroups supports the decision to classify them as a single exposure group.

## Discussion

This study identified significant disparities in prehospital and emergency care across a broad EMS cohort and two distinct populations requiring time-sensitive care: those with ischemic stroke and those with perforated ulcers. Patients with mental illness, substance use disorders, and dual diagnoses face challenges such as greater reliance on telephone advice in EMS, reduced access to reperfusion therapy, and delays in surgical intervention for specific subgroups. However, consistent adherence to guideline-based stroke care highlights areas where care quality remains equitable.

This study builds on prior research by the authors, which evaluated the quality of prehospital and emergency care across the same populations—EMS contacts, acute ischemic stroke, and emergency surgery—but focused exclusively on patients with mental illness stratified into three severity groups: minor, moderate, and major [17–19, 21–23]. In this study, we expanded the scope to include patients with substance use disorders and dual diagnoses. The inclusion of substance use disorders highlights their burden, excess mortality, stigma, and reliance on emergency services, providing a fuller view of disparities [1, 11, 12].

The significantly lower proportions of patients with substance use disorders and dual diagnoses in the stroke and emergency surgery populations than in the EMS cohort may reflect systemic barriers to accessing specialized care. Patients with lower social positions often rely on EMSs not only for emergencies but also for unmet social needs such as loneliness, isolation, or poverty [4, 24]. These populations also experience greater comorbidity burdens and systemic challenges, such as poor access to primary care and fragmented support systems [7, 8]. Stigma and diagnostic overshadowing further exacerbate these barriers, leading to missed opportunities to recognize serious underlying conditions [1, 2]. This dual reality underscores the need for EMSs to address both nonurgent utilization and ensure that genuine medical needs are not overlooked.

### Prehospital care and EMS response

Patients with mental illness, substance use disorders, or dual diagnoses were more likely to receive telephone advice rather than ambulance dispatch. For these patients, the EMS often serves as a primary point of contact owing to limited support in primary and social care settings. This reliance may reflect restricted primary care access, low organizational health literacy, and limited social support [1, 2, 5, 24]. Higher rates of repeat EMS contact and unplanned hospital visits further demonstrate gaps in care continuity for these populations. Wolfe et al. (2022) reported that fragmented care pathways and limited ongoing support often lead to frequent EMS recontacts among individuals with complex needs [4]. Similarly, Alsuhaibani et al. (2021) identified systemic barriers in dual-diagnosis care, where uncoordinated services drive reliance on emergency care for both acute and chronic unmet needs [3]. Addressing these challenges requires improving access to high-quality primary care and social services while strengthening emergency care. Solutions may include integrating social workers into EMS teams, as in assertive community treatment (ACT), which uses community-based teams to meet both mental health and social needs [12]. Targeted training for EMS staff to reduce stigma and improve communication, as recommended by Stone et al. (2021), [10] and embedding mental health expertise through models such as patient-centered medical homes (PCMHs) can increase care for vulnerable populations [13]. These integrated approaches improve care quality and reduce preventable reliance on EMSs [24].

### Acute stroke care

In acute stroke care, patients with mental illness, substance use disorders, and dual diagnoses are less likely to receive reperfusion therapy, a time-sensitive intervention critical for reducing stroke-related mortality and long-term disability. Other guideline-based treatments—such as imaging, stroke unit admission, and supportive therapies—were provided at significantly different rates across groups; however, these differences were small and unlikely to be clinically significant. This suggests that while protocols support high-quality care, barriers to accessing time-sensitive therapy remain. Prior research highlights stigma, diagnostic challenges, and delays in stroke response for these groups [2, 10, 22]. Expanding mental health training for EMSs and emergency staff may help address these barriers, ensuring timely and equitable access to life-saving therapies.

### Emergency surgery for perforated ulcers

For emergency surgery, patients with mental illness experienced significant delays in time-to-surgery for perforated ulcers, whereas delays were not observed for those with substance use disorders or dual diagnoses. While patients with mental illness experienced longer delays than did the reference group did, the overall median time to surgery for all patients exceeded national recommendations, highlighting systemic challenges in meeting time-critical surgical targets for perforated ulcers. Addressing these delays requires improvements in surgical pathways for all patients, with targeted strategies to ensure timely care for high-risk patients. Fewer days alive and out of hospital within 90 days postsurgery for patients with mental illness and substance use disorders reflect broader evidence of excess mortality and lost life years in these populations [8, 9]. Systemic delays in care likely contribute to both immediate and long-term outcomes.

### Limitations

This study has several limitations. While selection bias was minimized by using nationwide registries with high coverage, the generalizability of our findings may be limited. Specifically, patients with undiagnosed conditions or those receiving only telephone advice may not appear in registries, potentially excluding highly vulnerable populations who do not access or fully engage with emergency care.Our study design inherently includes only those who reached the hospital or reached out for the EMS, which may introduce index event bias—a form of selection bias where differences in healthcare access before hospital arrival could influence the observed disparities. This could underestimate the true burden of disparities for these groups. While the study's internal validity is strengthened by CPR-based follow-up, its generalizability may be further limited to healthcare systems like Denmark’s, where centralized registries and universal access to care are in place.

The classification of substance use disorders relies on hospital-based diagnostic codes and municipal treatment records, which may result in underdetection. If substance use is not directly relevant to the presenting issue, it may not be documented, leading to nondifferential misclassification. This misclassification likely dilutes the observed associations, producing conservative estimates of disparities. Given the higher healthcare needs of patients with substance use disorders, the true extent of inequities may exceed what our data suggest. Our findings should further be interpreted considering the heterogeneity within mental illness and substance use disorders. To account for this, we conducted subgroup analyses differentiating major vs. moderate mental illness and alcohol- vs. drug-related substance use disorders (see Supplementary Material). While our exposure classification aligns with prior research and supports robust statistical comparisons, future studies could further examine how specific psychiatric, and substance use disorders shape emergency care disparities.

While standardized registry data reduce information bias, variability in psychiatric diagnoses and manual data entry could introduce minor inaccuracies. Moreover, registry-based studies inherently lack the ability to capture social and clinical complexities affecting healthcare access, such as housing instability, social support, and health literacy. These unmeasured factors could influence disparities in care but were not directly assessed in our study.

The use of predefined quality metrics helps ensure consistency but may not fully capture important care dimensions, such as stigma or provider attitudes, which are recognized barriers to equitable emergency care [1, 2]. Qualitative research could complement our quantitative findings by providing deeper insights into these contextual factors. Although the large sample sizes provided high precision for most analyses, smaller subgroups (e.g., those with moderate mental illness) yielded wider confidence intervals, requiring cautious interpretation.

### Implications for practice and future directions

Addressing the disparities identified in this study requires a dual approach: improving primary care and social support services while strengthening emergency care for vulnerable populations. Models such as assertive community treatment (ACT) and patient-centered medical home (PCMH) demonstrate how integrating mental health and substance use expertise into care systems can enhance access, continuity, and quality of care [12, 13]. Similarly, embedding social workers within EMS teams and providing targeted training for EMS staff and telephone responders can help reduce stigma and improve communication [10, 23].

Ensuring adherence to existing clinical guidelines is essential to reducing disparities in emergency care. However, current guidelines may not fully account for the unique challenges faced by high-risk populations, including patients with mental illness, substance use disorders, and dual diagnoses. Tailored EMS protocols that address the unique needs of patients with mental illness and substance use disorders are essential such as enhanced screening, modified triage criteria, and multidisciplinary approaches to improve care coordination. These should include strategies to identify unmet care needs during initial contact and improve follow-up pathways to reduce repeat EMS contact and unplanned hospital visits [3, 4]. Moreover, prioritizing timely interventions in acute settings, such as reperfusion therapy for stroke and emergency surgery for perforated ulcers, is critical to minimizing delays and improving outcomes for high-risk groups.

## Conclusion

This study identifies significant disparities in prehospital and emergency care across three distinct populations: a broad EMS cohort and patients with time-critical conditions, ischemic stroke, and perforated ulcers. Patients with mental illness, substance use disorders, and dual diagnoses were more likely to receive telephone advice, experience repeat EMS contact and face lower rates of reperfusion therapy and delayed surgery. These disparities, alongside poorer outcomes such as higher poststroke mortality and fewer days alive and out of the hospital following surgery, highlight systemic challenges in meeting the needs of these vulnerable groups. However, areas of consistent care quality were observed, particularly in guideline-based stroke care, where treatments such as imaging, stroke unit admission, and supportive therapies were delivered at comparable rates across groups.

Achieving equitable outcomes may require further exploration of barriers such as stigma, fragmented care, and limited health literacy, as these factors are often associated with disparities in access and outcomes for vulnerable populations. While our findings highlight systemic gaps in emergency care, future research is needed to evaluate the effectiveness of tailored interventions and integrated care pathways in addressing these challenges and improving outcomes for patients with mental illness, substance use disorders, and dual diagnoses.

## Supplementary Information

Supplementary Material 1.

## Data Availability

Data cannot be shared publicly because of Danish legislation. Data can be accessed through the Danish Health Data Authority and Statistics Denmark for researchers at authorized institutions. Information on data access is available online (http://sundhedsdatastyrelsen.dk/da/forskerservice). Access to data requires approval from the Danish Data Protection Agency (https://www.datatilsynet.dk/english/legislation). The authors did not have special access privileges to these data.

## Abbreviations

ACT: Assertive Community Treatment
ASA: American Society of Anaesthesiologists (used in context of ASA score)
CI: Confidence Interval
CPR: Civil Personal Registration (Danish national ID system)
CT: Computed Tomography
EMS: Emergency Medical Services – (prehospital care provided by ambulance services and emergency call centres. This concerns all medical 112-calls in a Danish setting)
GP: General Practitioners
IQR: Interquartile Range
MR: Magnetic Resonance
PCMH: Patient-Centered Medical Home
RR: Risk Ratio
SUD: Substance Use Disorders
