# Supplementary material for: Nationwide study of emergency care quality for patients with substance use disorders and dual diagnoses across three distinct patient populations
Source: BMC Psychiatry. 2025 Mar 31;25:311. doi: 10.1186/s12888-025-06712-8 (PMC11959841; doi:10.1186/s12888-025-06712-8)
Supplement: Supplementary file 1 — Supplementary Material 1. [file 12888_2025_6712_MOESM1_ESM.docx]

**Supplementary Table 1: Co-variates: Definitions and data sources**

| **Variable** | **Definition** | **Data source** | |
| --- | --- | --- | --- |
| **Sex** | Defined as male or female according to the last digit in the civil registration number. Even numbers are used for women, odd numbers for men. If an individual changes gender (psychically and legally), they will be provided with a new number from the Danish Civil Registration System. | *The Danish Civil Registration System* is an administrative register established in 1968. It contains individual-level information on all persons residing in Denmark. It is updated daily with information on migration and vital status. The data in the Civil Registration System is virtually complete and have high accuracy, which allows for nationwide cohort studies with virtually complete long-term follow-up | |
| **Age** | Calculated based on the date of admission and date of birth registered in the Danish Civil Registration System. |  |  |
| **Death** | Date of death is available from the Danish Civil Registration System. |  |  |
| **Migration** | Based on data from the Danish Civil Registration System. |  |  |
| **Scandinavian stroke scale** | Defined as mild (score 45-58), moderate (30-44), severe (15-29), or very severe (0-14) | The Danish Stroke Registry | |
| **Hospital arrival time** | Time (date, hour, minute) of hospital arrival. If the patient was transferred from one hospital or one unit/department to another er hospital or unit/department, the admissions/contacts were merged, if there was less than 4 hours between the end of one contact to the beginning of the next. Arrival time was the time of the first hospital contact of the merged contacts. | *Danish Clinical Register of Emergency Surgery* and *Danish Stroke Registry (described in manuscript – methods section)* | |
| **Time: onset of symptoms** | Defined as time (date, hour, minute) of symptoms onset or “unknown” | *Danish Stroke Registry* | |
| **Presenting**  **symptom** | Based on “Danish Index for Emergency Care”, a criteria-based dispatch decision support tool. Each call is assigned a main symptom, which can be selected among 37 standardized symptoms, e.g., symptom card 26 “Impaired consciousness – paralysis – dizziness”. | *The Prehospital Database* | |
| **Level of**  **urgency** | Depending on the type and urgency of the symptoms (e.g., stroke symptoms, or breathing difficulties), the call is assigned a priority level from “A” to “E”. The highest priority level is urgency level A, corresponding to an  immediate response. | *The Prehospital Database* | |
| **Smoking status** | Defined as never smoker, previous smoker or current smoker | *Danish Clinical Register of Emergency Surgery*  *The Danish Stroke Registry* | |
| **Co-morbidity** | Defined as Charlson Comorbidity Index (CCI)^48^ based on diagnosis identified in the Danish National Patient Registry 10 years prior to admission. Grouped as: None (CCI 0), low (CCI 1–2), moderate (CCI 3-4) or high (CCI 5+) | | *The Danish National Patient Registry*  *The Danish National Patient Registry-Psychiatry* |
| **Co-habitation** | Defined as living alone or with someone else based on data from Statistics Denmark. Data was available for the status of co-habitation at December 31^st^ each year.  Data from the year of admission was used. If not available (e.g., death at the year of admission), data from the previous year was used. | *Statistics Denmark* is an extensive collection of administrative registers, containing individual-level data from governmental agencies. *The statistics on households and families* (FAM/FAIK) describe the total population living in Denmark. Daily deliveries from the Central Population Register (CPR) provide the basis for the statistics. Statistics are produced covering three different kinds of units: households, families and persons. The statistics describe these units based on household- and family-related variables (e.g. type of family, size of family, type of household). The basis for the statistics consists solely of CPR data on sex, age, marital status, references to spouses and parents, and address specification. The address data form the basis of the division into households, municipalities and regions. *The personal income statistics* (IND) only describes income for persons who are at least 15 years old at the end of the year and who are fully liable to pay tax in the year concerned. The a-income statistics mainly comprise of wages and transfers. The a-income amounts to 90 per cent of the total gross income. *The employment registry* RAS is an annually labor market statistic based on the population’s connection to the labor market on the last working day in November. *The Educational Attainment Register* (UDFF) gather information about the highest completed education for each single person based on the information in The Student Register and The Qualification Register. The primary data source to these statistics is the Student Register with data from 1974 onwards. | |
| **Income** | **Personal income** was defined as individual income at the year of admission. Personal income was grouped as “above national median”, “below national median” or “poverty” (defined as less than 50% of national median).  If income was not available the year of admission (e.g. death), the income of the previous year was used. |  |  |
| **Adherence to workforce** | **Individual adherence** to workforce was defined on the year of the admission. Adherence to workforce was grouped as: Working (incl. fulltime education), age-retirement or social subsidy. If not available at the year of admission due to e.g. death, data from the previous year was used |  |  |
| **Education** | Defined according to ISCED and the European consensus definitions as Low, Middle or High. |  |  |

**Supplementary Figure 1:** Directed Acyclic Graph (DAG)

To clarify our analytical approach, we used a Directed Acyclic Graph (DAG) to illustrate the relationships between exposure, outcome, confounders, mediators, and unmeasured variables. This approach helps justify our choice of covariates for adjustment and provides a transparent framework for understanding potential causal pathways.

- Exposure (Light Green): Mental illness, substance use disorders, and dual diagnoses.
- Outcome (Blue): Emergency care quality and clinical outcomes, including prehospital care metrics, reperfusion therapy for ischemic stroke, time-to-surgery for perforated ulcers, and mortality rates.
- Confounders (White): Age, sex, and comorbidities (via the Charlson Comorbidity Index).
- Stroke severity (measured by the Scandinavian Stroke Scale) was also treated as a confounder in stroke analyses, as it significantly affects clinical outcomes and emergency care quality. Although stroke severity could also act as a mediator if mental illness or substance use indirectly affects severity at admission (e.g., through delayed care-seeking), we adjusted for it to ensure consistency with existing literature.
- Mediators (Red): Socioeconomic factors, including income and education, and social factors such as cohabitation. These variables are likely influenced by the exposure and may, in turn, impact the outcome.
- **
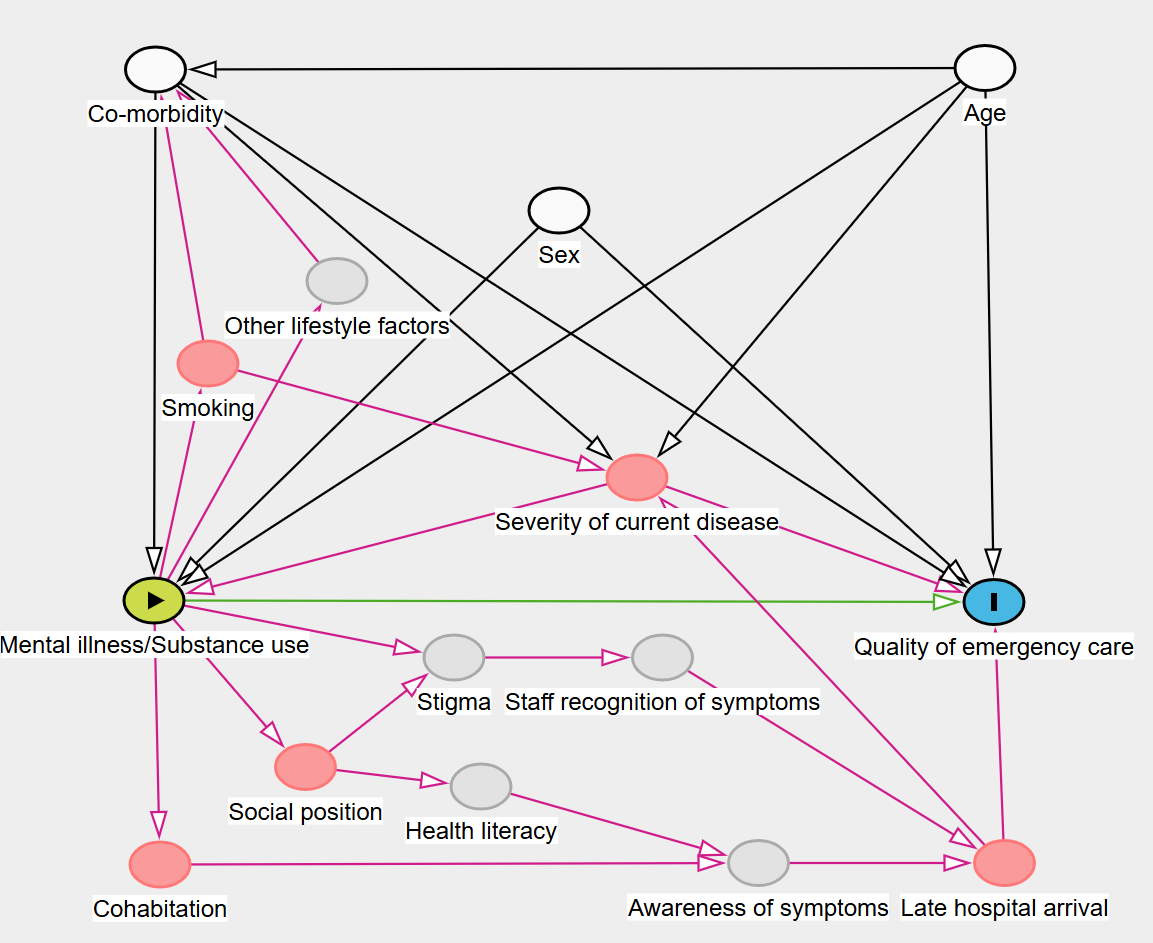
**Unobserved Variables (Grey): These include stigma, health literacy, lifestyle factors, and provider recognition of symptoms. These variables are relevant but were not directly measured or adjusted for in our models. Their influence remains a limitation of this study, as they may contribute to residual confounding.

**
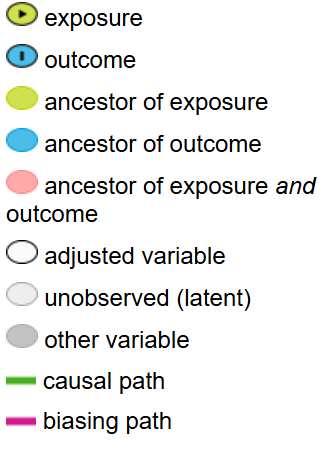
**

**Supplementary Figure 2: Sensitivity analyses for EMS measures with alternative definition of “mental illness”**

This analysis defined patients with mental illness as “major” mental illness (*within 10 years: A hospital-based diagnosis of schizophrenia spectrum disorders (DF20-22), bipolar disorder (DF30-31), or admission (>2 days) with depression (DF32-34) or emotionally unstable personality disorder (DF60.3)).*

Hence patients with “moderate” mental illness *(Any other psychiatric diagnoses (DF23-99) with or without admission or with any private psychiatrist consultation)* without substance use were included in the reference group. However, patients with moderate mental illness and substance use, were included in “dual diagnosis”.


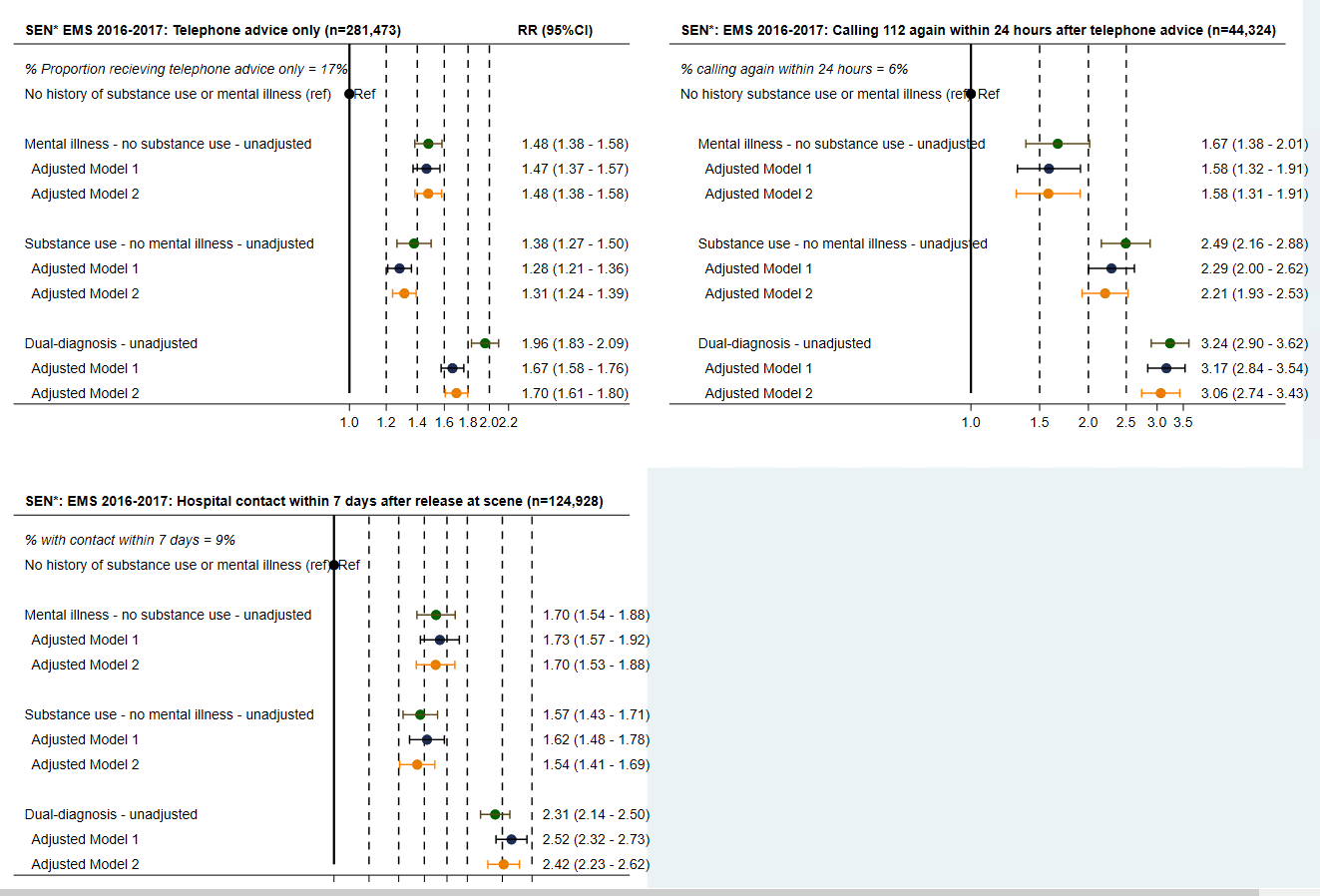


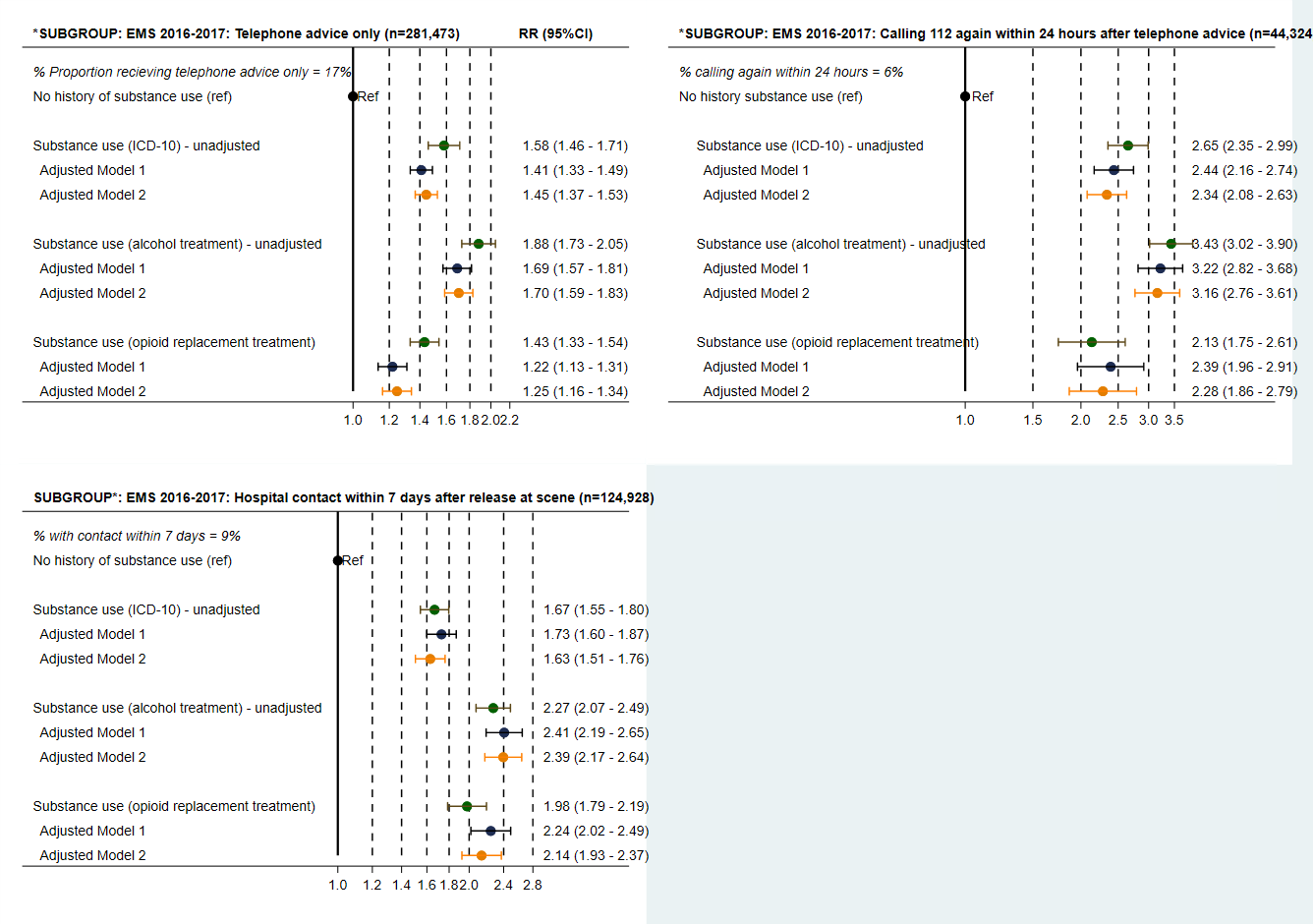
**Supplementary Figure 3: Sensitivity analyses for EMS measures with alternative definition of substance use:** Sensitivity analyses for EMS measures using alternative definitions of substance use, distinguishing between ICD-10 diagnoses, alcohol treatment, and opioid-replacement therapy. Results are presented for prehospital outcomes, as these demonstrated the most significant differences between groups and the greatest variation from the main analyses. These findings highlight the heterogeneity within the substance use group; however, given the consistently poorer outcomes across subgroups, we find it appropriate to classify them as a single exposure group, as done in the main article.
